# Epicardial Adipose Tissue Thickness and Ablation Outcome of Atrial Fibrillation

**DOI:** 10.1371/journal.pone.0074926

**Published:** 2013-09-16

**Authors:** Tze-Fan Chao, Chung-Lieh Hung, Hsuan-Ming Tsao, Yenn-Jiang Lin, Chun-Ho Yun, Yau-Huei Lai, Shih-Lin Chang, Li-Wei Lo, Yu-Feng Hu, Ta-Chuan Tuan, Hung-Yu Chang, Jen-Yuan Kuo, Hung-I Yeh, Tsu-Juey Wu, Ming-Hsiung Hsieh, Wen-Chung Yu, Shih-Ann Chen

**Affiliations:** 1 Division of Cardiology, Department of Medicine, Taipei Veterans General Hospital, Taipei, Taiwan; 2 Institute of Clinical Medicine and Cardiovascular Research Center, National Yang-Ming University, Taipei, Taiwan; 3 Division of Cardiology, Department of Internal Medicine, Mackay Memorial Hospital, Taipei, Taiwan; 4 Department of Medicine, Mackay Medical College and Mackay Medicine Nursing and Management College, Taipei, Taiwan; 5 Department of Health Industry Management, Kainan University, Taoyuan, Taiwan; 6 Division of Cardiology, National Yang Ming University Hospital, I-Lan, Taiwan; 7 Division of Radiology, Department of Internal Medicine, Mackay Memorial Hospital, Taipei, Taiwan; 8 Division of Cardiology, Department of Medicine, Cheng Hsin General Hospital, Taipei, Taiwan; 9 School of Medicine, Chung-Shan Medical University, Taichung, Taiwan; 10 Division of Cardiology, Department of Medicine, Wan-Fang Hospital, Taipei Medical University, Taipei, Taiwan; University of Illinois at Chicago, United States of America

## Abstract

**Objectives:**

Epicardial fat was closely related to atrial fibrillation (AF). Transthoracic echocardiography (TTE) has been proposed to be a convenient imaging tool in assessing epicardial adipose tissue (EAT). The goal of the present study was to investigate whether the EAT thickness measured on TTE was a useful parameter in predicting procedural outcomes of AF ablations.

**Methods and Results:**

A total of 227 paroxysmal AF (PAF) and 56 non-paroxysmal AF (non-PAF) patients receiving catheter ablations from 2008-2010 were enrolled. Echocardiography-derived regional EAT thickness from parasternal long-axis view was quantified for each patient. Free of recurrence was defined as the absence of atrial arrhythmias without using antiarrhythmic agents after ablations. The mean EAT thickness of the study population was 6.1 ± 0.8 mm. Non-PAF patients had a thicker EAT than that of PAF patients (7.0 ± 0.7 mm versus 5.9 ± 0.7 mm, p value <0.001). During the follow-up of 16 ± 9 months, there were 95 patients (33.6%) suffering from recurrences of atrial arrhythmias. Non-PAF, chads_2_ score, left atrial diameter and EAT thickness were independent predictors of recurrence after catheter ablations. At a cutoff value of 6 mm for PAF and 6.9 mm for non-PAF, the measurement of EAT thickness could help us to identify patients at risk of recurrences.

**Conclusions:**

EAT thickness may serve as a useful parameter in predicting recurrences after AF ablations. Compared to other imaging modalities, TTE can be an alternative choice with less cost and time in assessing the effects of EAT on ablation outcomes.

## Introduction

Atrial fibrillation (AF) is the most common sustained cardiac arrhythmia and has been associated with a marked morbidity, mortality, and socioeconomic burden [1,2]. As techniques and technologies have improved, catheter ablation of AF has become a standard and effective therapy for patients with symptomatic and drug-refractory AF, and its popularity continues to escalate [3]. Several recent studies have highlighted the close relationship between the amount of epicardial adipose tissue (EAT), presence of AF and recurrence of atrial arrhythmias after catheter ablation [4-6]. EAT represents a unique fat deposit because of its proximity to cardiac structures and its shared blood supply with the cardiac microcirculation [7]. Previous study has also demonstrated that it may serve as an abundant source of inflammatory mediators which directly damage the heart [8]. Although the measurement of the amount of EAT may provide useful information in managing AF patients, these imaging tools used before, such as cardiac computed tomography (CT) or magnetic resonance imaging (MRI), were expensive and subsequent processes of the images with off-line software were necessary to calculate fat volume. On the other hand, transthoracic echocardiography (TTE) is commonly performed in AF patients and can assess EAT quickly and accurately [9]. Recently, Lai et al. further showed that TTE-based measurement of EAT thickness highly correlated with that measured on CT [10]. Therefore, the purpose of the present study was to investigate whether TTE-derived EAT thickness was a convenient and useful parameter in predicting ablation outcome of AF.

## Methods

### Study population

Patients with symptomatic drug refractory AF who received radiofrequency catheter ablation for the first time under the guidance of a NavX mapping system (NavX, St Jude Medical, Inc., St. Paul, Minnesota) from 2008-2010 were studied. Those who didn’t receive regular follow up for at least 1 year after ablations were excluded. Finally, there were 227 paroxysmal AF (PAF) and 56 non-paroxysmal AF (non-PAF) patients enrolled in the present study. This study complies with the Declaration of Helsinki, and the study protocol was approved by the Institutional Review Board at Mackay Memorial Hospital, Taipei, Taiwan.

### Measurement of EAT thickness at TTE - the reproducibility and validation of accuracy

**Figure 1:**
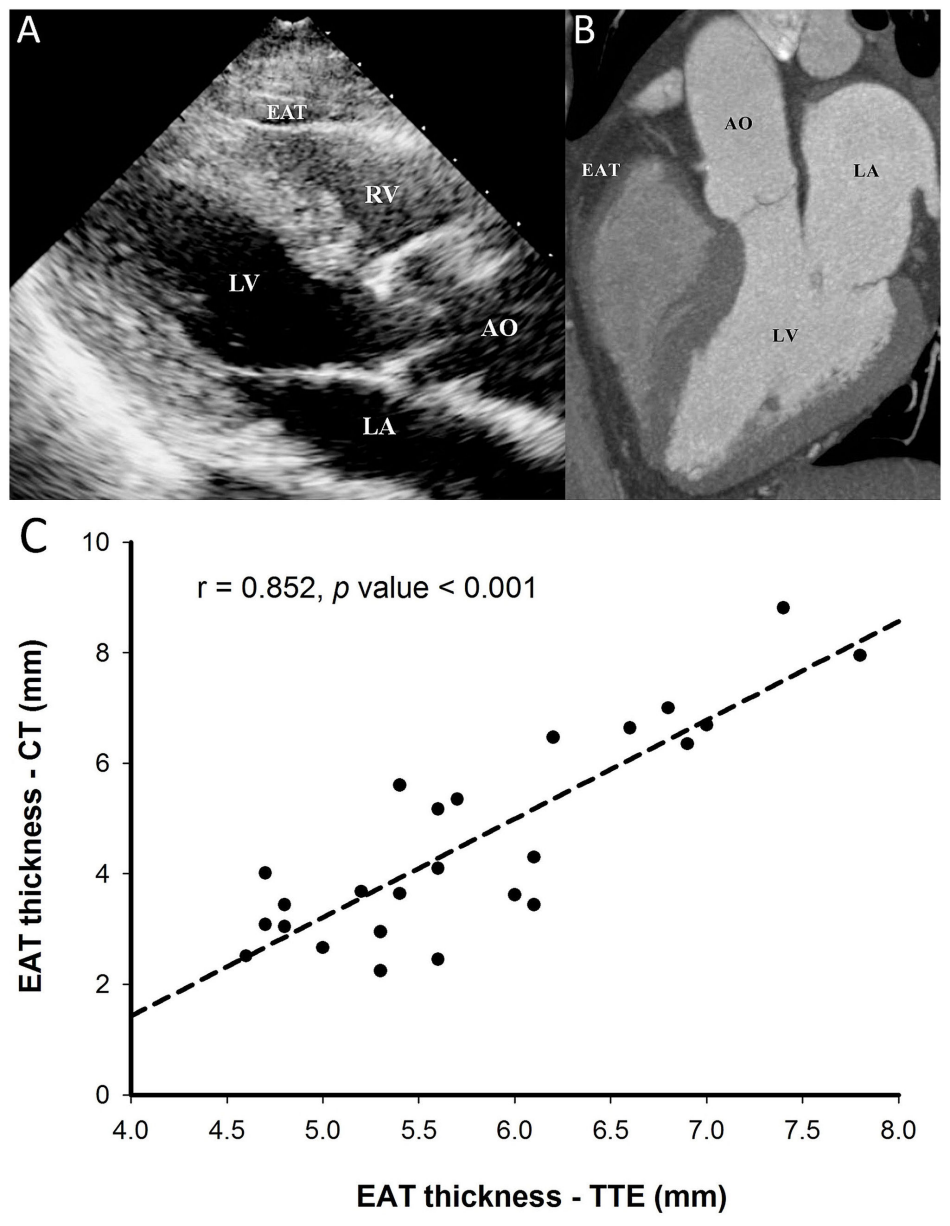
Measurement of EAT. The thickness of EAT measured in parasternal long-axis view of TTE (A) and 3-chamber view of CT with contrast enhancement (B) showed significant correlation (C). AO = aorta; CT = computed tomography; EAT = epicardial adipose tissue; LA = left atrium; LV = left ventricle; RV = right ventricle; TTE = transthoracic echocardiography.

Before catheter ablation, each subject underwent two-dimensional and M-mode transthoracic echocardiography using a Hewlett-Packard Sonos 5500 equipped with a 2.5-MHz to 4.5-MHz transducer. Epicardial fat is identified as the echo-free space between the myocardium and pericardium. At the parasternal long-axis view, EAT thickness was measured at the right ventricular free wall on M-mode cuts generated by placing the ultrasound beam perpendicular to the aortic annulus as proposed by Iacobellis et al. (Figure 1A) [11]. The measurements were performed by physicians who were blinded to the ablation outcomes retrospectively.

In order to confirm the reproducibility of the measurement, the EAT thicknesses of 120 study subjects (95 had PAF and 25 had non-PAF) were repeatedly measured by two physicians independently. The intraobserver and interobserver intraclass correlation coefficients for EAT thickness were 0.984 and 0.964, respectively (p value < 0.001). Using the Bland-Altman method [12], the mean difference between intraobservation and interobservation were 3.3% and 4.2%, respectively. Furthermore, the EAT thickness was assessed by contrast-enhanced CT among 25 subjects (15 had PAF and 10 had non-PAF) (Figure 1B) to validate the accuracy of TTE-based measurement of EAT for AF patients. The results of these 2 imaging tools showed significant correlation (r = 0.852, p value < 0.001) (Figure 1C).

### Correlations between EAT thickness and the volume of EAT

For the same 25 patients mentioned above, the volumes of total and peri-atrial EATs were measured by contrast-enhanced CT. The details about the measurements have been described in our previous publication [13]. The TTE-derived EAT thickness was significantly correlated with the volumes of total (r = 0.631, p value = 0.001) and peri-atrial (r = 0.591, p value <0.001) EATs.

### Catheter ablation of AF

Each patient underwent an electrophysiological study and catheter ablation in the fasting, non-sedative state after written informed consent was obtained. The details have been described previously [14-17]. In brief, after completing the left atrium geometry, continuous circumferential lesions were created encircling the right and left pulmonary veins (PVs) ostia guided by the NavX system using either a conventional 4-mm-tip or irrigated-tip catheter. The intention was to place the radiofrequency lesions at least 1–2 cm away from the angiographically defined ostia. Successful circumferential PV isolation was demonstrated by the absence of any PV activity or dissociated PV activity. If non-PAF did not stop after complete PV isolations, additional linear ablation was performed at both the anterior roof and lateral mitral isthmus. For PAF patients, the additional linear ablation was only performed when the AF was still inducible with programmed stimulation [15,18].

If non-PAF persisted after PV isolations and linear ablations, an additional complex fractionated electrographically guided substrate ablation was performed sequentially based on complex fractionated electrographic maps after PVI. Complex fractionated electrographic ablation was confined to the continuous complex fractionated electrograms (>5 seconds) in the left atrium (LA) and proximal coronary sinus [19,20]. The end point of complex fractionated electrographic site ablation was to obtain a prolongation of cycle length, eliminate complex fractionated electrograms, or abolish local fractionated potentials (bipolar voltage <0.05 mV).

After sinus rhythm was restored from AF by procedural AF termination or electric cardioversion, mapping and ablation were applied only to spontaneously initiating focal atrial tachycardias and non-PV ectopy that initiated AF. If any non-PV ectopy initiating AF from the superior vena cava was identified, isolation of the superior vena cava was guided by circular catheter recordings from the superior vena cava–atrial junction.

### Post-ablation follow up

After the catheter ablation, all patients received anti-arrhythmic drugs for 8 weeks to prevent any early recurrence of AF. Patients underwent regular follow-up (2 weeks after the catheter ablation, then every 1–3 months) at our cardiology clinic or with the referring physicians. During the follow-up, 24-hour Holter monitoring and/or cardiac event recording with a recording duration of one week were performed. The recurrence was defined as an episode of atrial arrhythmias lasting more than 30 seconds and confirmed by electrocardiograms 3 months after the ablation (blanking period). Freedom of recurrence was defined as the absence of atrial arrhythmias without using any antiarrhythmic agents after the catheter ablation. The long-term efficacy was assessed clinically on the basis of the clinical symptoms, resting surface 12-lead electrocardiogram, 24-hour Holter monitoring and/or 1 week cardiac event recordings.

### Statistical analysis

Differences between continuous values were assessed using an unpaired 2-tailed *t* test for normally distributed continuous variables, the Mann-Whitney test for skewed variables, and the chi-square test for nominal variables. A Cox regression analysis was used to identify the factors associated with recurrence. The optimal cut-off value of the EAT thickness in the prediction of recurrence was identified using the receiver operating characteristic (ROC) curve. The recurrence – free survival curve was plotted via the Kaplan-Meier method with the statistical significance examined by the log-rank test. All statistical significances were set at p value < 0.05 and all statistical analyses were carried out by SPSS 17.0 (SPSS Inc. USA).

## Results

### Baseline characteristics

**Figure 2:**
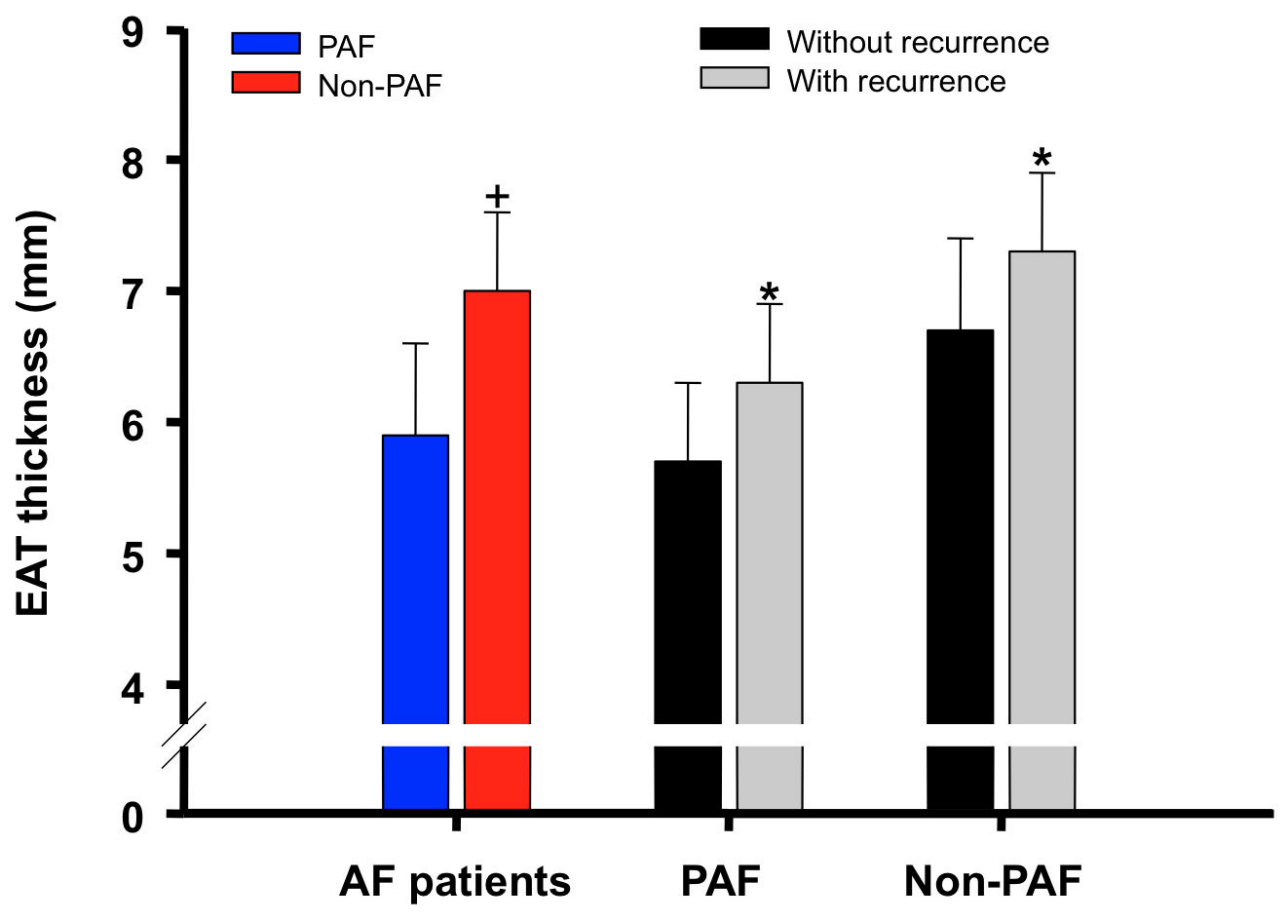
Differences of EAT thickness in PAF and non-PAF patients and those with and without recurrences. Increased EAT thickness was noted among non-PAF patients and those who experienced recurrences after catheter ablation. EAT = epicardial adipoes tissue; PAF = paroxysmal atrial fibrillation. +P value < 0.05, non-PAF versus PAF patients; *P value < 0.05, patients with recurrences versus patients without recurrences.

The baseline characteristics of the study population were shown in Table 1. The mean age of patients was 54.6 ± 10.4 years (range, 26-78 years), and 69.6% of them were male. Hypertension was the most common comorbidity, which was present in 44.2% of patients. The mean LA diameter was 39.4 ± 6.3 mm, and 45.9% of the study patients had a LA diameter > 40 mm. The mean EAT thickness was 6.1 ± 0.8 mm. Non-PAF patients had a thicker EAT than that of PAF patients (7.0 ± 0.7 mm versus 5.9 ± 0.7 mm, p value <0.001) (Figure 2).

**Table 1:** Baseline characteristics of the patients (n=283).

Variables	Study population, n = 283
Age, years	54.6 ± 10.4
Sex (male gender), %	69.6%
Non-paroxysmal AF, %	19.8%
History of AF, years	5.3 ± 3.4
Underlying diseases, %	
Hypertension	44.2%
Diabetes mellitus	16.3%
Congestive heart failure	7.8%
Coronary artery disease	12.0%
Previous stroke/transient ischemic attack	7.1%
Dyslipidemia	25.8%
CHADS_2_ score, median (inter-quartile range)	1 (0-1)
AADs use before ablation, median (inter-quartile range)	2 (1-3)
Body mass index, kg/m2	25.3 ± 3.6
Transthoracic echocardiogram	
LA diameter, mm	39.4 ± 6.3
LV wall thickness, mm	9.3 ± 1.0
LVEF, %	59.4 ± 7.2
Peak E-wave velocity (cm/s)	79.5 ± 20.7
Peak A-wave velocity (cm/s)	69.2 ± 41.1
E/A ratio	1.2 ± 0.5
EAT thickness, mm	6.1 ± 0.8
Recurrence of atrial arrhythmias, %	33.6%

AF = atrial fibrillation; AADs = antiarrhythmic drugs; EAT = epicardial adipose tissue, LA = left atrium; LV = left ventricle; LVEF = left ventricular ejection fraction

### Predictors of recurrence after catheter ablation

During a mean follow-up duration of 16 ± 9 months, there were 95 patients (33.6%) suffering from recurrences of atrial arrhythmias. The recurrence rates were 28.2% and 55.4% for PAF and non-PAF patients, respectively (p value <0.001). The factors associated with recurrences in the univariate and multivariate Cox regression analysis were shown in Table 2. Non-PAF, CHADS_2_ score, LA diameter and EAT thickness were identified to be independent predictors of recurrence after catheter ablation (Table 2).

**Table 2:** Cox regression analysis for predictors of recurrences.

Variables	**Univariateanalysis**			Multivariateanalysis^*^		
	HR	95% CI	P value	HR	95% CI	P value
Age (per year)	1.002	0.982-1.026	0.918	-	-	-
Sex (male gender)	1.234	0.781-1.949	0.368	-	-	-
Non-paroxysmal AF	2.884	1.869-4.448	<0.001	2.361	1.400-3.983	0.001
History of AF (pear year)	1.016	0.891-1.334	0.781	-	-	-
Coronary artery disease	1.165	0.985-1.534	0.148	-	-	-
CHADS_2_ score	1.321	1.197-1.548	<0.001	1.251	1.176-1.435	0.001
Body mass index (per kg/m^2^)	1.039	0.983-1.099	0.173	-	-	-
LA diameter (per mm)	1.070	1.032-1.109	<0.001	1.068	1.027-1.111	0.001
LV wall thickness (per mm)	0.942	0.752-1.179	0.601	-	-	-
Peak E-wave velocity (per cm/s)	1.005	0.994-1.016	0.400	-	-	-
Peak A-wave velocity (per cm/s)	1.002	0.997-1.007	0.366	-	-	-
E/A ratio	0.847	0.429-1.673	0.633	-	-	-
LVEF (per percent)	0.984	0.955-1.015	0.307	-	-	-
EAT thickness (per mm)	2.839	2.256-3.572	<0.001	2.863	2.112-3.882	<0.001

* The multivariate analysis included variables whose p values were <0.05 in the univariate model.

AF = atrial fibrillation; CI = confidence interval; EAT = epicardial adipose tissue; HR = hazard ratio; LA = left atrium; LV = left ventricle; LVEF = left ventricular ejection fraction

### EAT thickness and recurrence after catheter ablation

**Figure 3:**
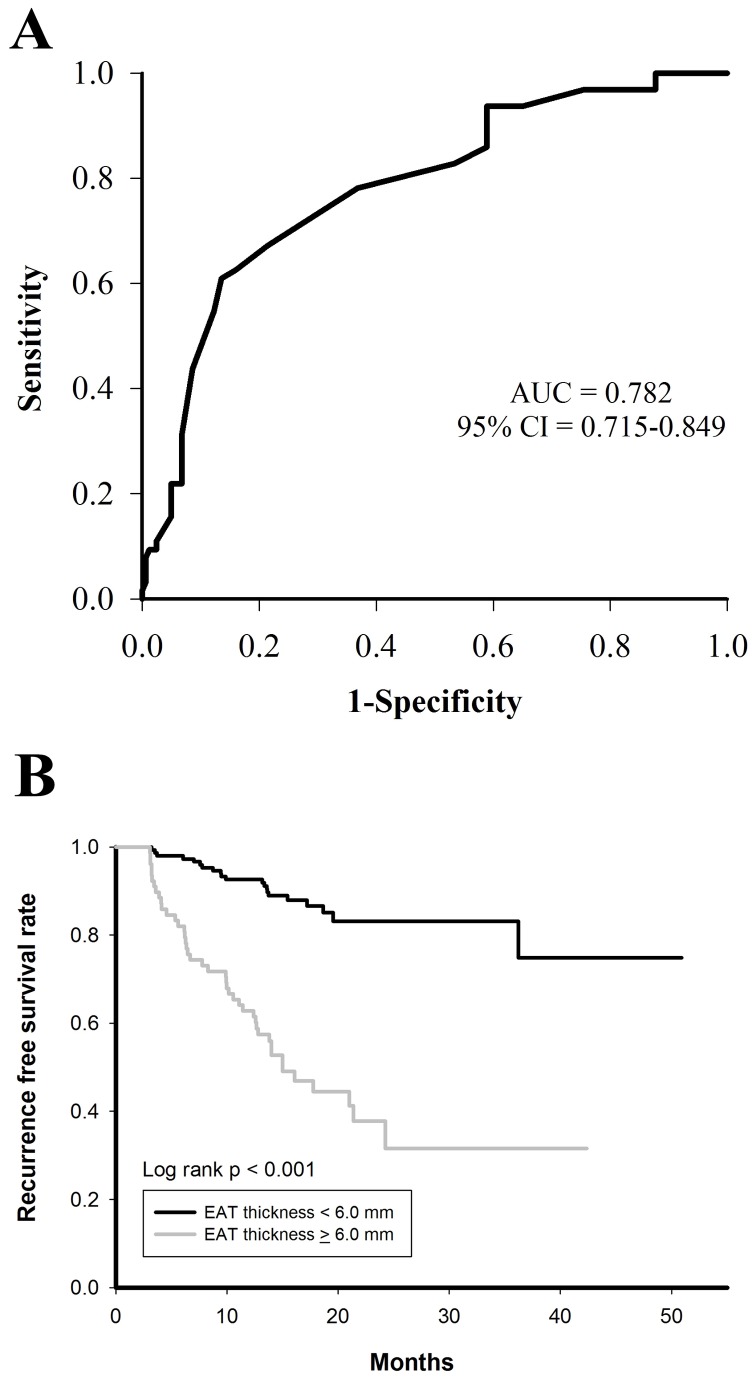
ROC curve and Kaplan-Meier analysis of EAT thickness in predicting recurrence after catheter ablation of PAF. At the cutoff value of 6.0 mm identified by the ROC curve (A), PAF patients with an EAT thickness of > 6.0 mm had a higher recurrence rate of atrial arrhythmias after catheter ablation (B). EAT = epicardial adipose tissue; PAF = paroxysmal atrial fibrillation; ROC = receiver-operator characteristic.

In the subgroup analysis for PAF and non-PAF patients, EAT was consistently thicker in patients with recurrences than those without (PAF: 6.3 ± 0.6 mm versus 5.7 ± 0.6 mm, p value <0.001; non-PAF: 7.3 ± 0.6 mm versus 6.7 ± 0.7 mm, p value = 0.001) (Figure 2). Figure 3A showed the ROC curve for predicting recurrences based on the EAT thickness among PAF patients (area under the curve = 0.782, 95% confidence interval = 0.715-0.849). At a cutoff value of 6.0 mm identified by the ROC curve, Kaplan-Meier survival analysis showed that patients with an EAT thickness of > 6 mm (sensitivity = 67.2%; specificity = 78.5%) were associated with a higher recurrence rate than those with an EAT thickness of < 6 mm (55.1% versus 14.1%, p value < 0.001) during the follow-up period (Figure 3B).

**Figure 4:**
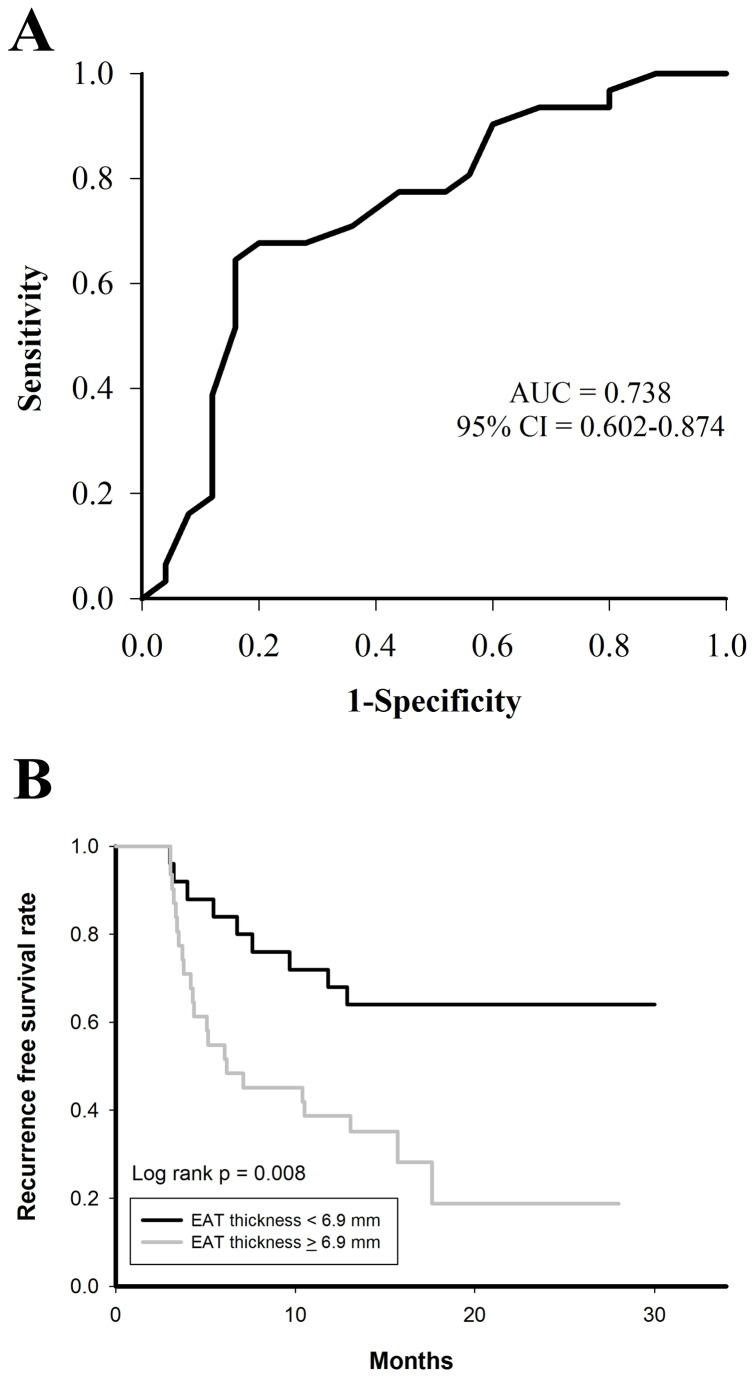
ROC curve and Kaplan-Meier analysis of EAT thickness in predicting recurrence after catheter ablation of non-PAF. At the cutoff value of 6.9 mm identified by the ROC curve (A), non-PAF patients with an EAT thickness of > 6.9mm had a higher recurrence rate of atrial arrhythmias after catheter ablation (B). EAT = epicardial adipose tissue; PAF = paroxysmal atrial fibrillation; ROC = receiver-operator characteristic.

The ROC curve based on EAT thickness in predicting recurrences after catheter ablation of non-PAF was shown in Figure 4A. At the cutoff value of 6.9 mm identified by the ROC curve (sensitivity = 71%; specificity = 64%), patients with thicker EAT had a higher recurrence rate after ablation procedures (71% versus 36%, p value = 0.008) (Figure 4B).

## Discussion

### Main findings

In this study, we investigated the associations between the TTE-derived EAT thickness and recurrences after AF ablations. The main findings were as follows: (1) EAT thickness assessed by TTE was significantly correlated with the volumes of total and peri-atrial EATs measured by cardiac CT. (2) EAT was thicker in non-PAF than PAF patients, which suggested that EAT was associated with AF chronicity and may play a role in AF progression(3). EAT thickness was a convenient parameter to predict recurrences after catheter ablation of AF.

### EAT, inflammation and AF

Obesity was recognized as a significant risk factor for AF [21,22], and the increasing amount of EAT among obese patients may explain for this clinical observation. Recently, there were more and more evidences demonstrating the close relationship between the epicardial fat and AF [4-6,23,24]. In these previous studies, increasing amount of EAT was reported to be associated with the presence of AF, AF chronicity and higher recurrence rate after AF ablations. EAT, a unique fat deposit, was directly contiguous with atrial and ventricular myocardium and highly metabolically active [8]. Tomasz et al. showed that EAT exhibited significantly higher levels of several inflammatory cytokines (interleukin-1, interleukin-6, and TNF-alpha) than subcutaneous fat [8]. Therefore, EAT serving as an abundant source of inflammatory mediators may predispose patients to AF by increasing the local inflammatory burden which directly damaged the atrium.

### EAT thickness and ablation outcomes

Increased regional EAT thickness measured by TTE was reported to be associated with increased left ventricular mass, metabolic syndrome, endothelial dysfunction, carotid atherosclerosis and coronary artery disease [25-29]. In the present study, we demonstrated that EAT thickness assessed by TTE was significantly correlated with the volumes of total and peri-atrial EATs. It could be an alternative convenient parameter to represent the volume of EAT and was useful in predicting AF ablation outcomes. Since the natural course after the ablation may differ between different types of AF [15,16], we identified the cut-off values of EAT thickness in predicting recurrences separately for PAF and non-PAF patients. At a cutoff value of 6 mm for PAF and 6.9 mm for non-PAF, the measurement of EAT thickness could help us to identify patients who were at risk of recurrences. What is the possible explanation for the usefulness of EAT thickness in predicting procedural outcomes of AF ablations? In the study performed by Lai et al. that enrolled 359 patients, increased EAT thickness was associated with an inflammatory status, represented by higher serum level of C-reactive protein (CRP) [10]. Since systemic inflammation was shown to be associated with an arrhythmogenic LA substrate and higher recurrence rate after AF ablations [30], the increased EAT thickness may represent increased inflammatory burdens which resulted in AF recurrences. The role of autonomic nervous system which resides in the EAT and even the effects of adipose tissue on impedance and current flow that may affect ablation depth are other possible mechanisms behind the link between the EAT and ablation outcomes. A further study is necessary to disclose the precise mechanisms.

### Comparisons with previous studies

Although increasing volume of literature has showed that EAT is significantly increased in AF patients [4,5,23,24], there were only several studies investigating the role of EAT in predicting recurrences after AF ablations [6,31,32]. Tsao et al. reported that abundance of EAT on cardiac CT was independently related to AF recurrences after ablations in 68 patients [31]. In the study performed by Nagashima et al. which measured EAT volumes with 3-dimensional reconstructed CT in 40 AF patients undergoing catheter ablation, greater EAT volume significantly predict AF recurrences [32]. More recently, Wong et al. performed an interesting study which enrolled a total of 110 AF patients and demonstrated that MRI-based measurement of EAT volume was a significant predictor of ablation outcomes [6]. There were several differences between the present study and previous reports. First, the present study enrolled a much larger number of patients than that of previous ones. Second, the cutoff values of EAT thickness in predicting recurrences for PAF and non-PAF patients were provided in the present study, and these data were lacking before. Third, the imaging tools (cardiac CT and MRI) used in previous studies were expensive and time consuming and, in the case of MRI, was contraindicated for patients who have received implantations of cardiac devices (i.e., permanent pacemaker). In contrast, TTE was commonly performed for AF patients in the daily practice, and can measure the EAT thickness conveniently and directly without additional processes of the obtained images.

### Clinical Applications

To the best of our knowledge, the present study was the first one to demonstrate that the TTE-derived regional EAT thickness may be helpful in identifying patients at risk of recurrences after AF ablations. For patients with a great EAT thickness and received catheter ablation, follow-up should be performed more closely to detect subtle recurrences. Besides, it deserves a further study to investigate whether these patients could obtain benefits from decreasing EAT amount through body weight reduction, regular exercises or the avoidance of high-fat diets.

### Study limitations

There were several limitations of the present study. First, we hypothesized that inflammation could be the possible mechanism for the association of the increased EAT thickness and higher recurrence rate after catheter ablations. However, the speculation was based on the results of previous investigations, and inflammatory markers, such as CRP and interleukin-6, were not available in the present study. Second, other components of body fat, such as visceral fat of abdomen, and waist circumference were not measured. These measurements may provide additional information on the effects of local versus systemic adiposity. Third, the cutoff values of EAT thickness in predicting recurrences were derived and tested in the same study population and it may exaggerate the effectiveness of the EAT thickness in predicting ablation outcomes. Lastly, the accuracy of the TTE-based measurement of EAT in predicting recurrences after AF ablations was not compared to that of CT or MRI-based measurements. However, the main purpose of the present study was to provide a convenient alternative method in evaluating the effects of EAT on ablation outcomes, rather than claiming that the EAT thickness assessed by TTE was better than that derived from other imaging tools.

## Conclusion

EAT in our study was shown to be a significant predictor of recurrences of atrial arrhythmias after catheter ablations of AF. Compraed with CT or MRI imaging, echocardiography is a reliable alternative imaging tool that requires less cost and time and may help us to identify patients who are at high risk of recurrences after ablations.
